# *Streptomyces*-Based Bioformulation to Control Wilt of *Morchella sextelata* Caused by *Pestalotiopsis trachicarpicola*

**DOI:** 10.3390/jof11060452

**Published:** 2025-06-13

**Authors:** Binghan Li, Yue Liu, Aihua Mao, Zhong Hu, Jin Li

**Affiliations:** 1 Key Laboratory of Southwest China Wildlife Resource Conservation, China West Normal University, Ministry of Education, Nanchong 637002, China; xhsfdxbinhan@126.com (B.L.); liuyue423520@163.com (Y.L.); 2 College of Life Sciences, China West Normal University, Nanchong 637002, China; 3 Department of Biology, College of Science, Shantou University, Shantou 515063, China; ahmao@stu.edu.cn (A.M.); hzh@stu.edu.cn (Z.H.); 4 Guangdong Provincial Key Laboratory of Marine Disaster Prediction and Prevention, Shantou University, Shantou 515063, China

**Keywords:** *M. sextelata*, *P. trachicarpicola*, *Streptomyces*, antagonism

## Abstract

In recent years, there has been extensive documentation of pathogenic fungi infecting *Morchella sextelata*. However, investigations of microorganisms with antagonistic properties against these pathogens are limited. This study successfully isolated two isolates of the genus *Streptomyces* (F16 and F19) from the rhizosphere soil of *M. sextelata* fruiting bodies, both of which exhibit potent antagonistic activity against *Pestalotiopsis trachicarpicola*, the causative agent of *M. sextelata* wilt disease. Comprehensive characterization, including physiological–biochemical tests and 16S rDNA sequence analysis, led to the identification of these isolates as *Streptomyces* sp. F16 and *Streptomyces* sp. F19. Both isolates significantly inhibited *P. trachicarpicola* through multiple mechanisms. The volatile compounds produced by these isolates effectively suppressed the conidial germination of *P. trachicarpicola* in vitro. Furthermore, fermentation filtrates at various dilutions exhibited pronounced antifungal activity against conidial germination, with *Streptomyces* sp. F16 showing 66.93% inhibition at a 50× dilution and *Streptomyces* sp. F19 achieving 49.22% inhibition under identical conditions. Field experiments have demonstrated the practical applicability of these antagonists. The topical application of fermentation filtrates (diluted 50×) from both isolates significantly reduced the incidence and severity of disease in *M. sextelata* cultivation. Notably, the yield improvements were substantial: fields treated with *Streptomyces* sp. F16 produced 299.6 g/m^2^, whereas those treated with *Streptomyces* sp. F19 yielded 277.65 g/m^2^. These yields significantly surpassed those of both the untreated control group (231 g/m^2^) and the *P. trachicarpicola*-inoculated group (134.93 g/m^2^). These findings indicate that the two isolates not only effectively control *P. trachicarpicola* but also increase the yield of *M. sextelata*.

## 1. Introduction

Fungi known as *Morchella* spp. are prized for their use in both culinary and medicinal applications, with the term ‘morels’ encompassing all species within this genus [1]. Morels are rich in amino acids, adenosine, and essential flavor compounds, and they also have high amounts of protein, lipids, dietary fiber, vitamins, minerals, and various bioactive nutrients [2]. Researchers have focused on the bioactive compounds found in morels, such as phenolics, ascorbic acid, and carotenoids, because of their strong antioxidant effects and related biological functions [3]. Owing to their high nutritional and commercial value, the indoor and outdoor cultivation of morels has garnered significant global attention. Since 2012, the application of external nutrients and the successful breeding of improved morel strains have enabled mainland China to produce 16,466 hectares outdoors during the 2021–2022 season [4]. However, with the rapid expansion of morel cultivation, diseases have emerged as the most significant threat to production, primarily caused by bacterial and fungal pathogens [5]. Recent studies have focused on fungal pathogens affecting morel cultivation, including stipe rot caused by *Fusarium* spp. [6], cobweb disease induced by *Cladobotryum* spp. [7], white mold disease attributed to *Paecilomyces penicillatus* [8], etc.

In recent years, morel wither diseases have caused severe outbreaks across multiple cultivation bases in China, with incidence rates reaching 80% [9]. It has been reported that the pathogens causing morels wither diseases include *Diploöspora longispora* [10], *Paecilomyces penicillatus* [8], and *Aspergillus* spp. [11]. Our recent research identified *Pestalotiopsis trachicarpicola* as the causative agent of apothecium wither in cultivated *Morchella sextelata* for the first time [12]. The disease primarily manifests on the pileus of morels, with rare occurrence on the stipe. Initial symptoms appear as irregular white fuzzy lesions on the fruit surface, which progressively expand to cause localized atrophy, desiccation, and perforation. Severe infections may lead to rot and wither of the pileus, accompanied by abnormal morphogenesis of the fruit [10]. Notably, the symptoms caused by different pathogens are slightly different. The pathogenic fungus *P*. *trachicarpicola* induces wither disease in *M*. *sextelata* through a two-stage infection process. During the initial phase, hyphal colonization results in the formation of white feathery lesions on the pileus surface, whereas in the second stage, mycelial webs spread along vertical and horizontal ridges, progressively penetrating the fruit parenchyma [12]. This leads to severe symptoms including desiccation, cracking, and pore formation on the pileus, ultimately resulting in large-scale yield reduction and significant economic losses for morels cultivators.

Actinomycetes, predominantly saprophytic aerobic microorganisms, are widely distributed in the environment and serve as important biocontrol agents with extensive applications. Actinomycetes are capable of producing a diverse array of high-value secondary metabolites, including β-lactams, peptides, glycopeptides, piperazines, polyketides, nucleosides, and other bioactive compounds [13]. These metabolites exhibit a wide spectrum of biological activities, such as antimicrobial, anticancer, immunosuppression, and insecticidal properties, making them critical resources for drug discovery and agricultural applications. The application of actinomycetes in the biocontrol of plant diseases has received more and more attention, with demonstrated efficacy against pathogens causing apple tree rot [14], bitter gourd wither [15], and cucumber wither [16]. However, research on biocontrol microorganisms that target fungal diseases of morels remains scarce, particularly with respect to the development of biocontrol strategies against *P. trachicarpicola* induced *M. sextelata* wither, a condition that currently lacks systematic investigation. In this study, actinomycetes from *M. sextelata* rhizoplane soil, which exhibited significant inhibitory activity against *P. trachicarpicola*, were isolated and subjected to preliminary investigations of their antagonistic mechanisms.

## 2. Materials and Methods

### 2.1. Screening of Antagonistic Actinomycetes

Both *P. trachicarpicola* and *M. sextelata* strains [12] were isolated and preserved by the Fungal Resources Research Section, Key Laboratory of Southwest China Wildlife Resource Conservation, China. Soil samples (at a 2–5 cm depth immediately below the stipe) were collected from 20 *M. sextelata* sporocarps, followed by serial dilution isolation using Gause’s No.1 medium (containing (per liter): soluble starch, 20 g; KNO_3_, 1 g; K_2_HPO_4_, 0.5 g; MgSO_4_·7H_2_O, 0.5 g; NaCl, 0.5 g; FeSO_4_·7H_2_O, 0.01 g; and agar, 20 g, with pH adjusted to 7.4–7.6 before sterilization) to screen for actinomycetes. These reagents were purchased from Sangon Biotech (Shanghai) Co., Ltd. (Shanghai, China). The antifungal activity of the isolated actinomycetes against *P. trachicarpicola* in *M. sextelata* was evaluated via the plate confrontation method. *P. trachicarpicola* disks (5 mm diameter) were transferred to the center of potato dextrose agar (PDA) medium plates, while actinomycetes (5 mm diameter) were transferred to two parallel sites 2.5 cm away from the *P. trachicarpicola*. A control group containing only *P. trachicarpicola* was established. All plates were incubated inverted at 28 °C. When the *P. trachicarpicola* mycelium in the control group had completely colonized the plate surface, mycelial growth inhibition in the experimental groups was assessed. The *P. trachicarpicola* colony diameters were measured, and antimicrobial activity was calculated using Formula (1) [17]. Three biological replicates were conducted per experimental group.

(1) $$The\;mycelium\;growth\;inhibition\;rate\left(\%\right)=\frac{A-B1}{A-B2}\times 100\%$$

where *A* is the colony diameter of the control *P. trachicarpicola*; *B*1 is the colony diameter of the experimental *P. trachicarpicola*; and *B*2 is the initial colony diameter of *P. trachicarpicola* (5 mm).

The isolated actinomycetes were cultured in Gause’s No.1 medium for fermentation at 28 °C for 6 days, and the fermentation supernatant was collected by centrifugation. A secondary-metabolite-enriched coculture medium was prepared by adding 10% (*v*/*v*) sterile fermentation supernatant to the PDA medium. *P. trachicarpicola* (5 mm diameter) was transferred to a coculture medium, followed by incubation at 28 °C in constant darkness. Sterile water was used as a control substitute for the fermentation supernatant. When *P. trachicarpicola* mycelium fully covered the plate in the control group, the experimental colony diameters were measured via the cross-hatching method [18]. Three biological replicates were conducted per group. The mycelial growth inhibition rate was calculated using Formula (1).

### 2.2. Identification of Antagonistic Actinomycetes

The screened antagonistic actinomycetes were cultured in Gause’s No.1 medium at 28 °C for 6 days. The morphological characteristics, including colony shape, pigmentation, and growth kinetics, were systematically documented. The basic physiological and biochemical characteristics of the isolated antagonistic actinomycetes were determined by referring to the bacterial identification method presented in the *Manual of Identification of Common Bacterial Systems* [19]. After culturing these isolates at 28 °C for 6 days, the biomass was subsequently harvested through centrifugation (12,000× *g*, 10 min), and genomic DNA was extracted using a commercial bacterial DNA extraction kit (Tiangen Biotech, Beijing, China). The 16S rRNA gene was amplified via PCR with primers 27F (5′-AGAGTTTGATCMTGGCTCAG-3′) and 1492R (5′-TACGGYTACCTTGTTACGACTT-3′). Amplification was carried out under the following conditions: 95 °C for 5 min (initial denaturation); 30 cycles of 95 °C for 1 min, 55 °C for 30 s, and 72 °C for 1 min 30 s; and a final extension at 72 °C for 10 min [20]. The PCR products were sent to Sangon Biotech (Shanghai) Co., Ltd., (Shanghai, China) for sequencing. The sequences were compared with known sequences in the National Center of Biotechnology Information (NCBI) database via BLAST+ 2.14.1. A phylogenetic tree was constructed via the neighbor-joining method with MEGA 7.0 software [21].

### 2.3. Assessment of Antimicrobial Efficacy of Volatile Compounds

Antagonistic actinomycetes (5 mm in diameter) were transferred to the center of PDA medium plates and incubated at 28 °C for 3 days. A new PDA plate was subsequently prepared by inoculating *P. trachicarpicola* (5 mm diameter). After the lids of the two plates had been removed, the two plates were joined using sealing film, sealed securely, and positioned with a *P. trachicarpicola* plate as the lower layer [22]. The coculture was maintained at 28 °C. A control group containing only *P. trachicarpicola* was established. When the control *P. trachicarpicola* mycelium had completely colonized the plate surface, the experimental colony diameters were measured via the cross-hatching method. Three biological replicates were conducted per group. The mycelial growth inhibition rate was calculated using Formula (1).

### 2.4. Suppression of Conidial Germination in P. trachicarpicola by Fermentation Supernatant

The fermentation supernatant of antagonistic actinomycetes was serially diluted (0-, 5-, 10-, 20-, 50-, and 100-fold) with sterile water. *P. trachicarpicola* conidium was transferred to each dilution and adjusted to a final concentration of 1 × 10^5^ cell/mL via a hemocytometer. Sterile water served as the negative control. The spore suspensions (25 μL) were spotted onto sterile microscope slides placed within 1% agar medium plates. The plates were incubated at 28 °C for 36 h. Spore germination was observed under a microscope, and each treatment was repeated five times. The germination inhibition rate was calculated via Formula (2).

(2) $$The\;germination\;inhibition\;rate\left(\%\right)=\frac{C-D}{C}\times 100\%$$

where *C* is the diameter of the *P. trachicarpicola* colony, and *D* is the diameter of the *P. trachicarpicola* colony after growth inhibition by antagonistic actinomycetes.

### 2.5. Biocontrol of the Fermentation Filtrate of Antagonistic Actinomycetes Against P. trachicarpicola in M. sextelata Fruiting Bodies

*M. sextelata* fruiting bodies (4–6 cm in height) were selected for the biocontrol test. After the fruiting bodies had been treated as follows, the infection was observed one week after growth. A 5 mm diameter hole was created on each fruiting body pileus. The following treatments were applied to each hole: Treatment 1: sterile water (25 μL) was added to each hole; Treatment 2: *P. trachicarpicola* spore suspension was inoculated into each hole; Treatment 3: *P. trachicarpicola* spore suspension was inoculated first, followed by the addition of 25 μL fermentation filtrate of antagonistic actinomycetes 24 h later; and Treatment 4: the fermentation filtrate of antagonistic actinomycetes was added first and allowed to air-dry for 2 h before the addition of *P. trachicarpicola* spore suspension. Each treatment test included 10 independent *M. sextelata* fruiting bodies.

### 2.6. Field Cultivation Assay

The field cultivation assay utilized fermentation filtrates of antagonistic actinomycetes at 5-, 10-, 20-, and 50-fold dilutions. The spores of *P*. *trachicarpicola* were aseptically transferred into fermentation filtrates of antagonistic actinomycetes at varying dilution levels using an inoculation needle. The mixture was vortex-mixed and adjusted to a final concentration of 1 × 10^5^ spore/mL using a hemocytometer. Sterile water served as the negative control. The addition protocols of each assay are shown in Table S1. Each assay received 167 g/m^2^ of *M. sextelata* and a 10 mL/m^2^ mixture. The duration of the *M. sextelata* field cultivation assay was approximately three months. The field trial was configured with optimized bed and ditch dimensions to maximize *M. sextelata* cultivation efficiency. Each cultivation bed measured 3 m in length and 1 m in width (3 m^2^ surface area), while associated drainage ditches were constructed with the following dimensions: 3 m length × 0.2 m width × 0.2 m depth. The experimental layout adopted a randomized complete block design comprising 5 replicate plots per treatment block, with each experimental unit standardized to 3 m^2^. In addition, the fields were covered with black plastic film to maintain the humidity and temperature. The growth and disease incidence were continuously monitored throughout the growth period of the fruiting bodies. Upon complete fruiting body maturation, the total harvest yields per treatment block were quantified using electronic scales. The average yield of *M. sextelata* per square meter (g/m^2^) was calculated for 5 replicates of each treatment group, and the yields of the different treatment groups were compared.

## 3. Results

### 3.1. Isolation of Antagonistic Actinomycetes

**Figure 1:**
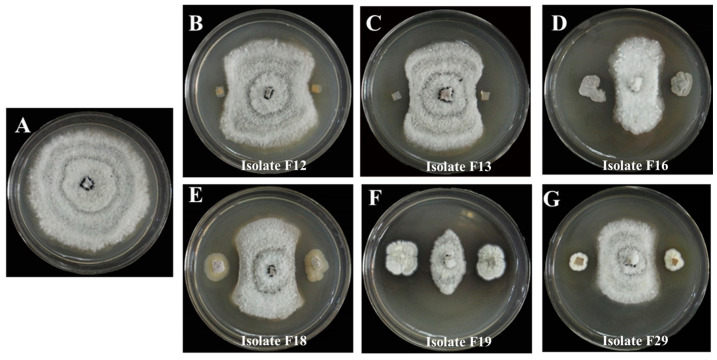
Inhibitory effects of the 6 isolates on mycelium growth of *P. trachicarpicola.* (**A**) *P. trachicarpicola*; (**B**) *P. trachicarpicola* and isolate F12; (**C**) *P. trachicarpicola* and isolate F13; (**D**) *P. trachicarpicola* and isolate F16; (**E**) *P. trachicarpicola* and isolate F18; (**F**) *P. trachicarpicola* and isolate F19; (**G**) *P. trachicarpicola* and isolate F29.

**Figure 2:**
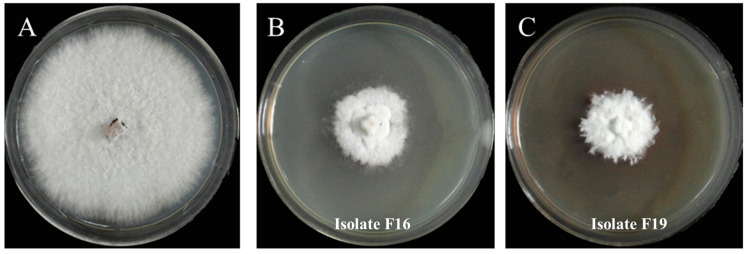
Inhibitory effects of fermentation filtrates of antagonistic isolates on *P. trachicarpicola*. (**A**) *P. trachicarpicola*; (**B**) *P. trachicarpicola* and the fermentation filtrates of isolate F16; (**C**) *P. trachicarpicola* and the fermentation filtrates of isolate F19.

A total of 29 colonies were isolated from the soil samples collected from the stipe of *M. sextelata* and preserved on Gao’s No. 1 slant medium. The isolates are numbered from F1 to F29. Using *P. trachicarpicola* as a test indicator species, six antagonistic isolates (F12, F13, F16, F18, F19, and F29) that exhibited significant inhibitory effects (antifungal rates > 50%) against *P. trachicarpicola* were initially identified through plate confrontation assays (Table S2). The highest antifungal rate among the six isolates was observed for isolate F19, reaching 80.59%, followed by isolate F16, with an antifungal rate of 70.04%. Figure 1 provides a clearer demonstration of the inhibitory effects of the six isolates on *P. trachicarpicola* growth. The inhibitory activity of the fermentation filtrates from the six antagonistic isolates was subsequently analyzed. The results revealed that the mycelium growth of *P. trachicarpicola* was significantly inhibited (Figure 2). Among the six isolates tested, the antifungal rates ranged from 19.61% to 62.54% (Table 1), with isolates F16 and F19 exhibiting antifungal rates exceeding 60%, which was statistically significant compared with those of the other isolates (*p* < 0.05). On the basis of these results, isolates F16 and F19 were selected as the dominant antagonistic isolates.

### 3.2. Identification and Biochemical Characteristics of Antagonistic Actinomycetes

**Figure 3:**
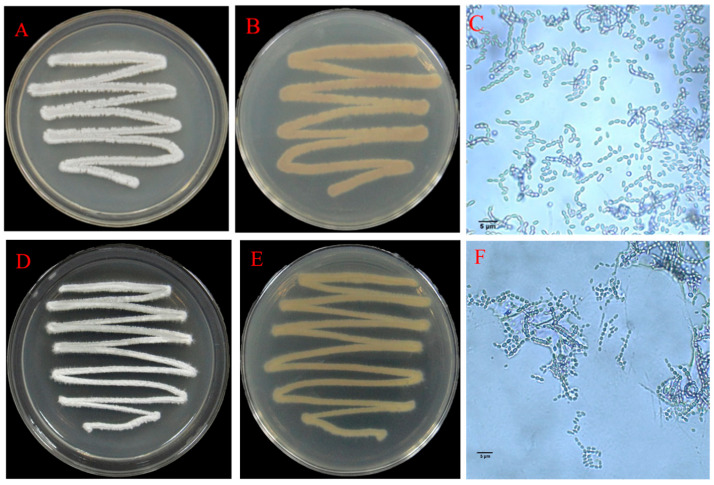
Morphology of colonies and spores of antagonistic isolates F16 and F19 (1000×). (**A**–**C**) Isolate F16; (**D**–**F**) isolate F19; (**A**,**D**) front of the medium; (**B**,**E**) reverse side of the medium; (**C**,**F**) spores.

As shown in Figure 3A–C, isolate F16 exhibited distinct morphological features on Gao’s No. 1 medium: Initially, the aerial mycelium was white, and the subsequent formation of spore masses resulted in a light purple color. The spores were elliptical with a smooth surface. In contrast, isolate F19 displayed similar initial white aerial mycelium but developed blue-gray spore masses upon maturation, producing elliptical spores without surface ornamentation (Figure 3D–F). The carbon and nitrogen source utilization analysis revealed that both isolates F16 and F19 utilized all 11 tested carbon sources and 4 nitrogen sources. It demonstrated the strongest utilization capacity for glucose, sucrose, glycerol and peptone, followed by moderate utilization of fructose, lactose, inositol, maltose, D-mannitol, KNO_3_, and (NH_4_)_2_SO_4_. The poorest utilization was observed for trehalose, starch, and cellulose (Table S3). Notably, neither isolate F16 nor isolate F19 exhibited hydrogen sulfide production or nitrate reduction activity. Furthermore, isolate F16 lacked gelatin liquefaction activity, whereas isolate F19 exhibited this capability, highlighting a notable distinction in metabolic functions between the two isolates.

**Figure 4:**
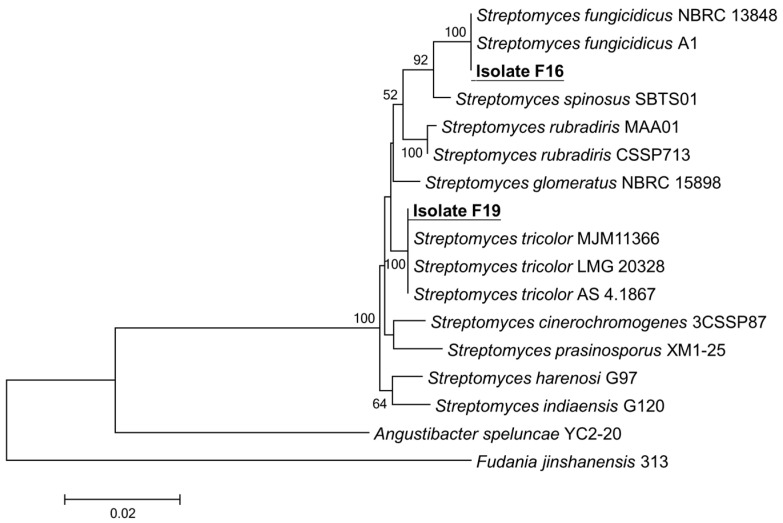
Phylogenetic tree of isolates F16 and F19 based on the 16S rRNA gene sequence.

After 16S rRNA gene sequencing was completed, the sequences of isolates F16 and F19 were compared with the known sequences in the NCBI database by using BLAST. Meanwhile, the GenBank accession numbers for the 16S rRNA gene sequence of isolates F16 and F19 were PV653593 and PV653594, respectively. The 16S rRNA gene sequences with high homology were downloaded and compared using MEGA 7.0 software to construct a phylogenetic tree by using the neighbor-joining method. The results revealed (Figure 4) that two isolates belong to the genus *Streptomyces*. Isolate F16 clustered with *Streptomyces fungicidicus* in a cluster, whereas isolate F19 clustered with *Streptomyces tricolor*. Finally, the two isolates were tentatively identified as *Streptomyces* sp. F16 and *Streptomyces* sp. F19.

### 3.3. Determination of Antifungal Effects of Volatile Substances from Isolates F16 and F19 Against P. trachicarpicola

**Figure 5:**
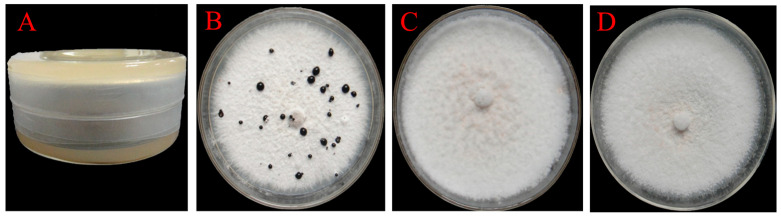
Inhibitory effects of volatile products of antagonistic isolates F16 and F19 against *P. trachicarpicola*. (**A**) Schematic of dual-plate co-culture assay; (**B**) *P. trachicarpicola* control; (**C**) *P. trachicarpicola* cultured with antagonistic isolate F16; (**D**) *P. trachicarpicola* cultured with antagonistic isolate F19.

When *P. trachicarpicola* was cocultured with isolates F16 and F19 under various conditions, *P. trachicarpicola* presented consistent average growth rates across all of the tested cultures, completely covering the agar surface by day 8. However, notable differences were observed in colony morphology on day 14: while the control group produced conidia (Figure 5B), no conidiation was detected in the coculture systems with *Streptomyces* sp. F16 (Figure 5C) or *Streptomyces* sp. F19 (Figure 5D). These observations suggest that both antagonistic isolates may secrete volatile compounds that specifically inhibit conidiation in *P. trachicarpicola*.

### 3.4. Determination of the Inhibitory Effects of Fermentation Filtrates of Isolates F16 and F19 Against P. trachicarpicola

**Figure 6:**
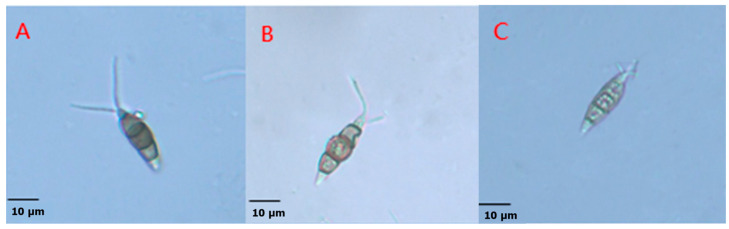
The effect of 5-fold dilution of fermentation filtrate on the conidial morphology of *P. trachicarpicola*. (**A**) CK; (**B**) the fermentation filtrates from *Streptomyces* sp. F16; (**C**) the fermentation filtrates from *Streptomyces* sp. F19.

The experimental investigation of the inhibitory effects of fermentation filtrates from *Streptomyces* sp. F16 and *Streptomyces* sp. F19 at various dilutions on the conidial germination of *P. trachicarpicola* revealed significant morphological alterations in treated conidia, including pronounced swelling and an abnormal morphology (Figure 6). The fermentation filtrates of *Streptomyces* sp. F16 and *Streptomyces* sp. F19 potently suppressed *P. trachicarpicola* conidial germination when diluted 5-fold. Notably, both isolates demonstrated concentration-dependent inhibition, with 50-fold dilutions achieving inhibition rates of 66.93% and 49.22%, respectively. However, when the mixture was diluted 100-fold, the inhibitory effects were substantially reduced, resulting in inhibition rates of only 36.96% and 21.21%, respectively (Table 2). These results suggest that the antifungal compounds secreted by *Streptomyces* F16/F19 are likely the cause of inhibited germination, and highlight that antifungal activity significantly decreases with an increase in dilution.

### 3.5. Field Experiment for Evaluating the Efficacy of the Isolates F16 and F19 on P. trachicarpicola in M. sextelata

**Figure 7:**
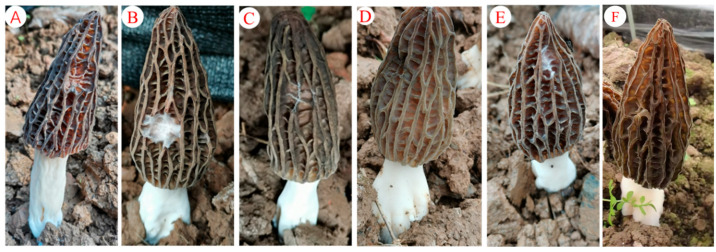
Inhibitory effects of fermentation filtrates of antagonistic isolates of *P. trachicarpicola* on *M. sextelata* fruiting bodies. (**A**) Sterile-water treatment; (**B**) inoculate with only *P. trachicarpicola*; (**C**) *Streptomyces* sp. F16 fermentation filtrate 24 h after inoculation with *P. trachicarpicola*; (**D**) *P. trachicarpicola* inoculated 2 h after *Streptomyces* sp. F16 fermentation filtrate was applied; (**E**) *Streptomyces* sp. F19 fermentation filtrate applied 24 h after inoculation with *P. trachicarpicola*; (**F**) *P. trachicarpicola* inoculated after *Streptomyces* sp. F19 fermentation filtrate was applied for 2 h.

The preventive effects of fermentation filtrates from the antagonistic isolates *Streptomyces* sp. F16 and *Streptomyces* sp. F19 against *P. trachicarpicola* on *M. sextelata* fruiting bodies were investigated. The in vitro inhibition rates of the two isolates against *P. trachicarpicola* reached 89.57% and 75.64%, respectively. (1) Preventive efficacy of *Streptomyces* sp. F16 on *M. sextelata* fruiting bodies: When *P. trachicarpicola* was inoculated onto fruiting bodies followed by the application of *Streptomyces* sp. F16 filtrate 24 h later, no disease symptoms were observed compared with those in the control group (Figure 7C). These results show that the method significantly inhibited the growth of *P. trachicarpicola*. The application of *Streptomyces* sp. F16 filtrate 2 h before *P. trachicarpicola* inoculation also completely prevented infection, with *P. trachicarpicola* growth inhibited under the fruiting skin (Figure 7D). (2) Comparative efficacy of *Streptomyces* sp. F19: Similarly to *Streptomyces* sp. F16, *Streptomyces* sp. F19 filtrate application 24 h after inoculation partially reduced disease severity, although limited lesion formation was still observed on fruiting bodies compared with the control (Figure 7E). Pretreatment with *Streptomyces* sp. F19 filtrate 2 h before infection effectively suppressed *P. trachicarpicola* colonization (Figure 7F), albeit with weaker protective effects than those of *Streptomyces* sp. F16. Both fermentation filtrates demonstrated antifungal activity against *P. trachicarpicola* on *M. sextelata* fruiting bodies. Notably, *Streptomyces* sp. F16 exhibited superior efficacy in both pre- and postinoculation treatments, achieving complete disease prevention and robust *P. trachicarpicola* suppression, whereas *Streptomyces* sp. F19 showed partial protection under specific conditions. These results highlight the potential of isolate-specific fermentation products for developing targeted biocontrol strategies against *M. sextelata* wilt disease.

**Figure 8:**
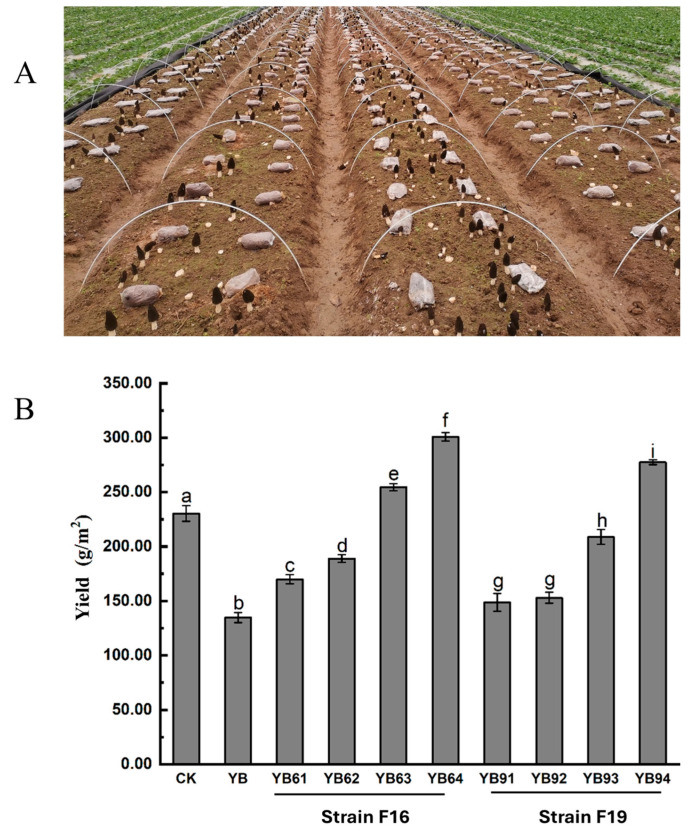
Effects of two antagonistic isolates on yield of *M. sextelata*. (**A**) Cultivation fields of *M. sextelata*; (**B**) yield of *M. sextelata* in different test fields. Superscript lowercase letters (a, b, c…) were assigned to denote statistically significant differences (*p* < 0.05) in mean yields among test fields.

Field cultivation experiments were carried out according to Table S1. Field observations revealed that the experimental field YB presented significantly fewer *M. sextelata* primordia formations than did the control field (CK). During the early fruiting stage, young fruiting bodies in the experimental field YB were highly susceptible to *P. trachicarpicola* invasion. Notably, the experimental groups treated with 20-fold and 50-fold diluted fermentation filtrates presented increased primordium counts relative to those in the experimental field YB. However, the immature ascocarps in these groups remained vulnerable to infection. In contrast, the experimental groups receiving 5-fold and 10-fold dilutions not only presented increased primordium formation but also presented substantially reduced infection rates in young fruiting bodies (Figure 8B). These results indicate the concentration-dependent efficacy of fermentation filtrates in promoting primordium development while suppressing pathogen infection during critical growth stages.

Field observations demonstrated that inoculation with *P. trachicarpicola* alone resulted in a significant reduction in *M. sextelata* yield. The yields of the experimental fields YB61, YB62, YB91, and YB92 were intermediate, exceeding those of the *P. trachicarpicola*-inoculated field YB but remaining lower than those of the noninoculated control group (field CK). These findings suggested that 20-fold and 50-fold dilutions of the fermentation filtrate from antagonistic isolates exhibited limited antifungal efficacy. In contrast, the experimental fields treated with 5-fold or 10-fold dilutions of *Streptomyces* sp. F16 or 5-fold dilutions of *Streptomyces* sp. F19 surpassed both the experimental field YB yield and the CK yield, indicating exceptional productivity (Figure 8B). Notably, the 5-fold diluted filtrates from *Streptomyces* sp. F16 and *Streptomyces* sp. F19 yielded 299.6 g/m^2^ and 277.65 g/m^2^, respectively, which were significantly greater values than the value of 231 g/m^2^ observed in the *M. sextelata*-alone group (CK).

## 4. Discussion

A series of physical control methods for *Morchella* wilt disease, including field-based prevention, pest attraction and elimination, the timely removal of contaminated nutrient bags, and the prompt removal of infected fruiting bodies, have shown limited efficacy. With the increasing focus on sustainable agricultural practices, biological control has emerged as a promising alternative [23]. Actinomycetes are ubiquitous microorganisms that inhabit diverse ecological niches such as soil, air, plant debris, and sedimentary environments [24]. The ability of the *Streptomyces* genus to produce a diverse array of secondary metabolites is a well-established characteristic within the actinomycetes order. The secondary metabolites present antibacterial properties against diverse plant diseases [25]. For example, *Streptomyces lunalinharesii* was isolated from the rhizosphere soil of *Illicium verum* and exhibited significant antifungal activity against *Colletotrichum gloeosporioides*, the causative agent of *I. verum* anthracnose [26]. Similarly, *Streptomyces olivoreticuli* screened from the rhizosphere soils of plants had a strong antagonistic effect on *Rhizoctonia solani*, which is a soilborne pathogen that causes root rot in various crops [27].

Extensive studies have demonstrated that strains of the genus *Streptomyces* exhibit significant suppressive effects against diverse plant pathogens, indicating great potential for the development of eco-friendly antifungal strategies targeting *Morchella* diseases [28]. In this work, we isolated 29 actinomycetes from the rhizosphere soil of *M. sextelata* through a multistage screening process. Initially, plate confrontation assays and fermentation filtrate rescreening identified antagonistic actinomycetes with significant antifungal activity against *P. trachicarpicola*. Subsequent characterization on the basis of morphological features, culture patterns, physiological and biochemical tests, and 16S rRNA gene sequences confirmed that two isolates, isolates F16 and F19, belong to the *Streptomyces* genus.

The plate confrontation assays revealed that two antagonistic strains, *Streptomyces* sp. F16 and *Streptomyces* sp. F19, produce antimicrobial substances capable of significantly inhibiting *P. trachicarpicola* mycelial growth. To elucidate whether these substances are soluble or volatile, we systematically investigated the effects of fermentation filtrates and volatile compounds from the two isolates. The results demonstrated that (1) the fermentation filtrates suppressed *P. trachicarpicola* mycelial growth through soluble antimicrobial components, and that (2) the volatile compounds inhibited the conidial germination of *P. trachicarpicola*. This dual-mode inhibition mechanism aligns with findings by Sudha et al., who reported that volatile metabolites from *S. rochei* ASH disrupted fungal hyphal structures, achieving inhibition rates of 63.75% and 68.52% against *Fusarium moniliforme* and *Curvularia lunata*, respectively [29].

Spores are resilient to harsh environmental conditions and can germinate once favorable conditions are restored [30]. Therefore, evaluating the inhibitory effects of antagonistic actinobacteria on *P. trachicarpicola* spore germination is critical for disease control [31]. In this work, fermentation filtrates of *Streptomyces* sp. F16 and F19 significantly impaired *P. trachicarpicola* conidial growth, resulting in swelling and rupturing of the spores. These results align with previous research on *Streptomyces* species [32], which demonstrated similar conidial inhibition patterns through secreted antimicrobial compounds.

*Streptomyces* strains can further alleviate disease pressure by stimulating elements of the plant immune system and increasing plant productivity [33]. On the basis of field yield, 5- and 10-fold dilutions of the fermentation filtrates from both strains not only suppressed *P. trachicarpicola* growth but also significantly promoted an increase in *M. sextelata* yield. Similar research results show that *Streptomyces flavovirens* can be used to increase mushroom yield and contribute to disease control against green mold disease caused by *Trichoderma aggressivum* [34]. This dual functionality suggests that these strains exhibit mycelial-growth-promoting effects alongside *P. trachicarpicola* inhibition. Therefore, *Streptomyces* can be used not only as a biological control agent for pathogens of *M. sextelata*, but also as a growth promoter for *M. sextelata*.

## 5. Conclusions

Overall, this study provides basic data for the accurate identification of the causative agents and prevention and control of diseases affecting *M. sextelata* in the field. *Streptomyces*-based bio-formulation has emerged as the most effective strategy for decreasing disease severity and enhancing fruit yield in *M. sextelata* cultivation. These results provide valuable insights into sustainable agricultural practices, suggesting that integrating biocontrol agents into crop management can lead to improved disease resistance and yield stability in the face of environmental challenges.

## Figures and Tables

**Table 1:** Antifungal rates of 6 isolates with antagonistic activity.

Isolate Number	Colony Diameter (cm)	Antifungal Rate (%)
CK	8.42 ± 0.08 a	—
F12	6.47 ± 0.15 b	24.66% ± 0.02 a
F13	6.87 ± 0.18 c	19.61% ± 0.02 b
F16	3.47 ± 0.15 d	62.54% ± 0.02 c
F18	6.45 ± 0.15 b	24.87% ± 0.02 a
F19	3.65 ± 0.14 e	60.23% ± 0.02 d
F29	4.82 ± 0.12 f	45.50% ± 0.01 e

Note: Lowercase letters (a, b, c…) after the numbers were assigned to denote statistically significant differences (*p* < 0.05).

**Table 2:** Effects of different dilution ratios of fermentation filtrate from isolates F16 and F19 on the conidial germination of *P. trachicarpicola*.

Dilution Times	Inhibition Rate of Conidial Germination (%)	
	*Streptomyces* sp. F16	*Streptomyces* sp. F19
0	99.81% ± 0.00 a	97.86% ± 0.01 a
5	99.22% ± 0.01 a	94.94% ± 0.01 a
10	88.91% ± 0.02 b	79.96% ± 0.01 b
20	78.40% ± 0.03 c	66.73% ± 0.03 c
50	66.93% ± 0.05 d	49.22% ± 0.03 d
100	36.96% ± 0.03 e	21.21% ± 0.04 e

Note: the lowercase letters (a, b, c…) after the numbers were assigned to denote statistically significant differences (*p* < 0.05).

## Data Availability

The original contributions presented in this study are included in the article and Supplementary Materials. Further inquiries can be directed to the corresponding author.

## Supplementary Materials

The following supporting information can be downloaded at: https://www.mdpi.com/article/10.3390/jof11060452/s1. Table S1: Inoculation methods of each test group in the field; Table S2: Inhibitory effect of primary screening of actinomycetota strains against *P. trachicarpicola*; Table S3: Biochemical characteristics of antagonistic isolates F16 and F19.

## Abbreviations

The following abbreviations are used in this manuscript:

PDA	Potato dextrose agar medium
NCBI	National Center of Biotechnology Information
