# Supplementary material for: Streptomyces-Based Bioformulation to Control Wilt of Morchella sextelata Caused by Pestalotiopsis trachicarpicola
Source: J Fungi (Basel). 2025 Jun 13;11(6):452. doi: 10.3390/jof11060452 (PMC12193963; doi:10.3390/jof11060452)
Supplement: Supplementary file 1 [file jof-11-00452-s001.zip › jof-3684547-supplementary.pdf]

Streptomyces-Based Bioformulation to Control Wilt of *Morchella sextelata* Caused by *Pestalotiopsis trachicarpicola*

Binghan Li [1,2], Yue Liu [1,2], Aihua Mao [3], Zhong Hu [3] and Jin Li [1,2,4,*]

Table S1 Inoculation methods of each test group in the field

Test name	Planting method
CK	*M. sextelata*
YB	*M. sextelata* + *P. trachicarpicola* spore suspension
YB91	*M. sextelata* + *P. trachicarpicola* spore suspension + the fermentation supernatant of isolate F19 at 50-fold dilution
YB92	*M. sextelata* + *P. trachicarpicola* spore suspension + the fermentation supernatant of isolate F19 at 20-fold dilution
YB93	*M. sextelata* + *P. trachicarpicola* spore suspension + the fermentation supernatant of isolate F19 at 10-fold dilution
YB94	*M. sextelata* + *P. trachicarpicola* spore suspension + the fermentation supernatant of isolate F19 at 5-fold dilution
YB61	*M. sextelata* + *P. trachicarpicola* spore suspension + the fermentation supernatant of isolate F16 at 50-fold dilution
YB62	*M. sextelata* + *P. trachicarpicola* spore suspension + the fermentation supernatant of isolate F16 at 20-fold dilution
YB63	*M. sextelata* + *P. trachicarpicola* spore suspension + the fermentation supernatant of isolate F16 at 10-fold dilution
YB64	*M. sextelata* + *P. trachicarpicola* spore suspension + the fermentation supernatant of isolate F16 at 5-fold dilution

Table S2 Inhibitory effect of primary screening actinomycetota strains against *P. trachicarpicola*

Strain number	Colony diameter (cm)	Antifungal rate (%)
CK	8.40±0.10	—
F1	7.30±0.20	13.92%±0.03
F2	6.03±0.15	29.96%±0.01
F3	6.13±0.06	28.69%±0.01
F4	7.40±0.20	12.66%±0.03
F5	5.57±0.21	35.86%±0.03
F6	6.00±0.10	30.38%±0.01
F7	5.57±0.15	35.86%±0.02
F8	6.07±0.15	29.54%±0.02
F9	7.30±0.10	13.92%±0.01
F10	4.70±0.17	46.84%±0.02
F11	5.17±0.15	40.93%±0.02
F12	4.43±0.15	**50.21%±0.02**
F13	4.23±0.06	**52.74%±0.01**
F14	5.77±0.12	33.33%±0.01
F15	6.17±0.06	28.27%±0.01
F16	2.87±0.15	**70.04%±0.02**
F17	5.77±0.06	33.33%±0.01
F18	3.43±0.21	**62.87%±0.03**
F19	2.03±0.12	**80.59%±0.01**
F20	5.70±0.10	34.18%±0.01
F21	6.47±0.06	24.47%±0.01
F22	7.37±0.06	12.24%±0.01
F23	7.13±0.15	16.03%±0.02
F24	7.00±0.17	13.08%±0.01
F25	6.57±0.15	23.21%±0.02
F26	5.87±0.06	32.07%±0.01
F27	6.17±0.15	28.27%±0.02

F28	6.87±0.06	19.41%±0.01
F29	3.50±0.10	**62.03%±0.01**

Table S3 Biochemical characteristics of antagonistic isolates F16 and F19

Biochemical characteristics	Isolate F16	Isolate F19
**Carbon source**		
Fructose	++	++
Glucose	+++	+++
Lactobiose	++	++
Sucrose	+++	+++
Inositol	++	++
Trehalose	+	+
Glycerinum	+++	+++
Maltobiose	++	++
D- mannitol	++	++
Starch	+	++
Cellulose	+	+
**Nitrogen source**		
Peptone	+++	+++
KNO_3	++	++
$(NH_4)_2SO_4$	++	++
Urea	+	++
Gelatin liquefaction	-	+
H_2S reduction	-	-
Nitrate reduction	-	-
